# Experimental testing of fracture fixation plates: A review

**DOI:** 10.1177/09544119221108540

**Published:** 2022-08-03

**Authors:** Shiling Zhang, Dharmesh Patel, Mark Brady, Sherri Gambill, Kanthan Theivendran, Subodh Deshmukh, John Swadener, Sarah Junaid, Laura Jane Leslie

**Affiliations:** 1 Aston Institute of Materials Research (AIMR), Aston University, Birmingham, UK; 2 Invibio Biomaterial Solutions Limited, Hillhouse International, Thornton-Cleveleys, UK; 3 Sandwell and West Birmingham Hospital NHS Trust, Birmingham, UK

**Keywords:** Tribological testing, fracture fixation, wear loss, debris generation, morphology, fretting, biomedical devices, biomaterials, trauma plate, debris characterisation

## Abstract

Metal and its alloys have been predominantly used in fracture fixation for centuries, but new materials such as composites and polymers have begun to see clinical use for fracture fixation during the past couple of decades. Along with the emerging of new materials, tribological issues, especially debris, have become a growing concern for fracture fixation plates. This article for the first time systematically reviews the most recent biomechanical research, with a focus on experimental testing, of those plates within ScienceDirect and PubMed databases. Based on the search criteria, a total of 5449 papers were retrieved, which were then further filtered to exclude nonrelevant, duplicate or non-accessible full article papers. In the end, a total of 83 papers were reviewed. In experimental testing plates, screws and simulated bones or cadaver bones are employed to build a fixation construct in order to test the strength and stability of different plate and screw configurations. The test set-up conditions and conclusions are well documented and summarised here, including fracture gap size, types of bones deployed, as well as the applied load, test speed and test ending criteria. However, research on long term plate usage was very limited. It is also discovered that there is very limited experimental research around the tribological behaviour particularly on the debris’ generation, collection and characterisation. In addition, there is no identified standard studying debris of fracture fixation plate. Therefore, the authors suggested the generation of a suite of tribological testing standards on fracture fixation plate and screws in the aim to answer key questions around the debris from fracture fixation plate of new materials or new design and ultimately to provide an insight on how to reduce the risks of debris-related osteolysis, inflammation and aseptic loosening.

## Introduction

A bone fracture is a crack or break in the bone which could be caused by traumatic incidents such as falls, accidents, sports and/or by pathological reasons where bones are weakened due to underlying health conditions, such as osteoporosis or bone cancer/tumours.^1^ Fracture fixation secures the broken bone segments in the desired position for healing to take place. Various fixation constructs can be adopted depending on the seriousness and the location of the fracture. One of the most commonly used fixation methods is the use of internal plates and screws that may be made of different biocompatible materials. The ultimate goal of a trauma fixation solution is to bring pre-injured functions back to patients in as short a time as possible, as well as avoiding any complications and side effects during or after surgery.

However, tribological wear around the fracture fixation construct (plates and screws) can occur and lead to adverse effects to the body.^2^ The consensus is that the high coefficient of friction between the implant components themselves and between them and the body tissues, along with the formation of debris adjacent to the implants, can lead to complications, including but not limited to inflammation, osteolysis, implant loosening, hypersensitivity and toxicity/carcinoginity.^3,4^ It is worth noting that besides tribology-induced debris, catastrophic plate failure may also generate debris, which contributes to the biological response. In addition, tribological characterisation is an aspect that is gaining increasing attention from various stakeholders such as patients, clinicians, medical device companies and regulatory bodies. It is a major factor in controlling and determining the long-term clinical performance of the fracture fixation plate within the implanted body. Moreover, advancements in healthcare and medical technology have increased the longevity of human beings and imposed ever-high demands on the mechanical and tribological characteristics of fracture fixation plates.

Nevertheless, research into this area of tribology in fracture fixation constructs is limited, especially in comparison with joint replacement applications. This is because the majority of the plates and screws are made from metal materials, which have long been adopted ever since the first introduction of internal fixation plates and screws.^5,6^ There was no tribological testing of the metal required at the time due to limited understanding of its impact and the impression that plates and screws would only stay in vivo for a relatively short period time, therefore tribology was not thought to be a concern. The emerging body of research in tribology within joint replacements over the last two decades have highlighted the potential harm metal debris have on the body.^2,4,7^ This sets a precedent for other implants such as fracture fixation constructs to re-evaluate the importance of tribology factors in current standard testing protocols. The aim of this literature review is to explore the current status on the testing methods of fracture fixation plates and identify some of the gaps and challenges.

## Methodology

**Figure 1.**
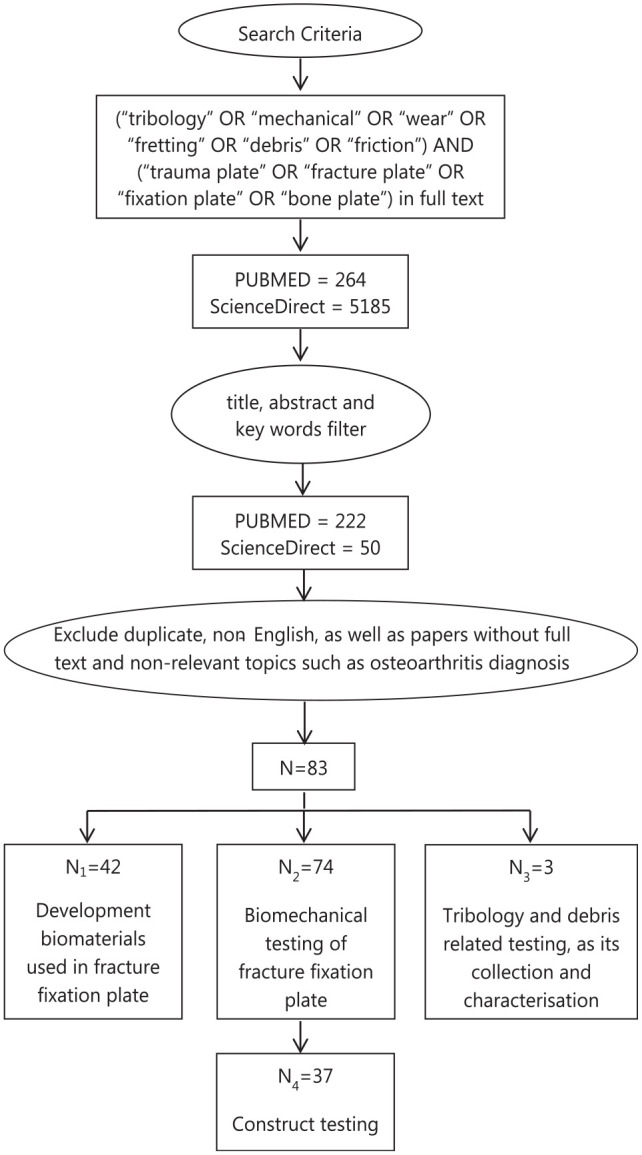
Flowchart showing the search and filtration criteria; a total of 83 papers were included in this review, among which there is an overlap of 35 papers between biomaterials development and biomechanical testing, an overlap of one paper between biomechanical testing and tribology studies.

Available publications on biomechanical testing of fracture fixation plates, particularly on tribological testing, were considered within this review paper. Figure 1 shows the search and filtering criteria for the selected publications to review. Firstly, papers were searched within PubMed and ScienceDirect databases, using the search criteria: (‘tribology’ OR ‘mechanical’ OR ‘wear’ OR ‘fretting’ OR ‘debris’ OR ‘friction’) AND (‘trauma plate’ OR ‘fracture plate’ OR ‘fixation plate’ OR ‘bone plate’).

About 264 papers within PubMed and 5185 papers within ScienceDirect were selected. The title, abstract and key words of these papers were then further checked, after which 222 PubMed papers and 50 ScienceDirect papers were left. Duplicate, non-English papers, as well as papers without full text and non-relevant papers such as osteoarthritis diagnosis were then further excluded, leaving 83 papers for review.

Those 83 papers to review were divided into three groups, among which 42 papers were regarding new biomaterials in fracture fixation plates, 74 papers were related to biomechanical testing where 37 papers were more related to experimental testing and three papers were about further tribological characterisation including debris characterisation and its biological impact. The overlaps between biomaterials development with biomechanical testing and tribology testing were 35 and 1, respectively. To this end, a review of the current ASTM and ISO standard on product testing of fracture fixation plate were also conducted.

It is worth noting that this may not have covered every published paper within the topic, nevertheless, it is broad enough to represent the current state-of-play in understanding and development of the tribological characterisation of fracture fixation plates. In addition to tribological tests, some construct tests that generate debris will also be considered due to the risk of catastrophic failures, particularly within relatively young patients.

## Development of biomaterials used in fracture fixation plates

Fracture fixation plates and screws are deployed as an internal fixation method that is intended not only to reduce the fracture, but also provide sufficient immobilisation to allow for the bone to heal.^8,9^ Starting from this point of view, the early understanding was that the fracture fixation plates shall be sufficiently stiff to provide a stable construct to hold the fractured bones in place. There are two main mechanisms of bone healing; primary healing and secondary healing, of which secondary healing accounts for the majority of bone healing and requires relative flexibility to achieve. Therefore, from the perspective of bone healing, the plate must not be too stiff, otherwise it will result in stress shielding where the load is borne mainly by the plate rather than the underlying bone tissue.^8,9^ As a result, a lack of controlled micromotion and compressive loading at the fracture site inhibits callus formation which negatively impacts the quality of the healed bone or leads to non-union.

Historically the first internal fixation plate was a metal plate invented by Lane in 1895.^5,6^ Since then, there has been a series of improvements in the metal that has been used, as well as the plate design. Metal. still remains the dominant material in fracture fixation plates and based on the current market usually consist of stainless steel and commercially pure titanium as well as its alloy.

Stainless steel, exhibiting a Young’s modulus of around 200 GPa,^10^ can provide sufficient strength to the fixation construct of plate and screws. Meanwhile, stainless steel also demonstrates corrosion resistance, especially SS 316L (ASTM F138) which is the most widely used stainless steel in orthopaedic implants including fracture fixation plates. The ‘L’ in SS 316L represents extra low carbon content (0.03 wt. %). Lower carbon will generate lower amounts of chromium carbide at the grain boundaries of the polycrystalline structure, which leads to better corrosion resistance. In addition, the low cost of stainless steel makes it affordable and promotes its adoption. However, SS 316L contains 13%–15% nickel which is potentially toxic and may also cause allergic reactions in patients with metal sensitivity.^11^ New nickel free stainless steel has been developed mainly to address this issue, though it is not yet widely clinically adopted.^11^

It is important to note that the stainless steel still does not have optimum corrosion resistance. To improve the anti-corrosion performance, titanium and its alloys (Young’s modulus of 110 GPa) started being used for internal fracture fixation after they became commercially available in the 1950s.^10^

Commercially pure titanium (CP Ti) refers to unalloyed titanium with minor amounts of impurity elements, such as C, O and Fe. There are four grades of CP Ti used for medical applications (ISO 5832-2), amongst which CP2 and CP4 are the most widely used for internal fixation plates.^12^ Titanium alloys, such as Ti6Al4V and Ti6Al7Nb are also used mainly due to their increased mechanical strength over CP Ti. In addition, research also shows that the corrosion resistance of Ti is also improved as a result of the introduction of the harmless elements, Al, V, Nb into pure titanium.^10,12^ What is more, despite being more expensive, clinical research demonstrated better bone quality after healing when using titanium plates because of the lower stiffness with modulus in comparison with stainless steel plates. The resulting lower stiffness was thought to reduce the stress shielding effect by lowering the stiffness discrepancy between cortical bone and metal plate.

Novel design concepts using additive manufacturing to 3D print porous plates from biomaterials such as 316 L, Ti and Ta is another area of development with the promise of improving properties and customisation to match the patient and therefore better union.^13,14^ Nonetheless the shortcomings of metal plates are becoming more and more noticeable due to the increasing performance requirements. Stress shielding is one of the most well reported drawbacks of metal plates.^15^ This is because according to Wolff’s law and Frost’s theory,^16^ when the plate is stiffer with higher Young’s modulus, it can prohibit secondary healing through callus formation and bone remodelling. The metallic fracture fixation plate is also associated with the release of metallic ions into the patient; on a small scale due to the uniform passive dissolution resulting from the slow diffusion of metal ions through the passive film, and on a larger scale, due to the breakdown of the passivity as a consequence of chemical (pitting and crevice corrosion) or mechanical (fretting corrosion) events.^17^ Besides metallic ions, debris is generated due to corrosion while the plate is functioning within the patient. It can also be generated by the relative motion at interfaces within the fixation construct and between the fixation construct and the adjacent bone or tissue. The ions and debris released into the patients can evoke host tissue responses and be detrimental to the patient.^18^ It is thus one of the most important factors that affects the performance of the fracture fixation plate. Another shortcoming is the lack of radiolucency of the metallic plates which can be an obstacle when it comes to assessing the healing of the bone, and also poses an additional challenge with oncological patients or patients who require radiotherapy.^19,20^

Due to the above pitfalls of stainless steel and titanium plates, efforts have been devoted into new biomaterial development around biodegradable materials and materials with less stiffness to reduce the stress shielding effect, such as polymers and composites.

Polymers such as polymethyl-methacrylate (PMMA), poly glycolic acid (PGA), L-poly-lactic-acid (PLLA), D-poly-lactic-acid (PDLA), poly ether–ether-ketone (PEEK), have widely been studied for bone fracture fixation applications. The Young’s modulus of PMMA, PGA, PLLA and PEEK is within the range of 3–4 GPa, which is similar to that of cancellous bone.^10,21,22^ Theoretically,^16^ these materials may reduce stress shielding during the bone healing process, nevertheless, they only have limited applications in dental implants and internal fixators such as spine cages and bone cement.^12^ The main obstacle for a wider application of polymer plates in fracture fixation is their poor mechanical properties.

As a result, composite materials, such as nanofiller reinforced high density polyethylene (HDPE) and carbon fibre composites,^23–28^ are explored to improve the strength of the polymer, where ceramics, metal and fibres are added. Among these, the most clinically developed composite is carbon fibre reinforced PEEK (CFR-PEEK), which is a composite made of continuous or discontinuous carbon fibres embedded in a PEEK matrix.^20,29,30^ Research and clinical studies demonstrate; greater callus formation^31^; 360° fracture visibility radiographically^30,32–34^; no metallic ion release and hence no adverse inflammation or other adverse biological responses related with metallic ions from the plate.

However, despite the potential advantages, there is also one main question raised, that is, the changes in mechanical behaviour of the fracture fixation construct in the development of these relatively new materials. These changes are likely linked into the micromotion between components and the subsequent nature of debris generation, which may lead to the adverse effect and failure of the implants. Furthermore, the stiffness disparity between non-metal plates and metal screws would need to be studied, not only from a mechanical perspective but also in reviewing micromotion at the fracture through the biotribology lens, which may also have a local or possibly systemic effect.

## Experimental testing of fracture fixation plates

**Figure 2.**
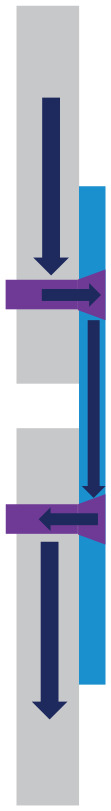
Schematic diagram showing the load transfer in fracture fixation construct.

Figure 2 shows a schematic diagram of a typical fracture fixation construct where locking plate and screws are deployed. The screw head locks into the plate providing both axial and angular stability. The contact surfaces within this construct contains surfaces between bone and screw, screw and plate and bone and plate. The load is transferred from bone, to screw, to plate, to screw and eventually back to bone. Relative motion between those contacting subjects can be caused by human movement in one form or another. One of the main outcomes from this micromotion is debris generation.

Among the 83 reviewed papers, 74 papers describe biomechanical testing of fracture fixation plates. Of these, 37 focus on experimental testing, and this is summarised in Table 5.

For biomechanical testing a common method that researchers have adopted to simulate a fracture is to generate a gap in either synthetic bones, simulated bones (computational models) or normal bones such as equine or human cadaver bones. From the 37 papers, the proportion of researchers using synthetic, simulated and natural bones were 38%, 31% and 31%, respectively, as demonstrated in Table 1. Natural cadaver bones would be the best physiological option to achieve more realistic and trustworthy data. It is highly required for regulatory purposes but comes with high associated cost. It could also be a challenge to achieve repeatable results due to the variation from the donor. Improving the test quantity could address this issue but requires access to a large quantity of cadaveric bones as well as even higher cost. Synthetic bones with homogeneous properties and simulated bones are therefore widely accepted as a cost saving method.

**Table 1.** Bone substrates adopted among studies on the construct testing (*n* = 37).

Gap size, mm	Number of studies	Proportion (%)
0	4	10
1	7	18
2	1	3
3	4	10
4	5	13
5	4	10
6	1	3
10	10	25
13	1	3
20	1	3
25	1	3
60	1	3
Total	40 (Three papers investigated two gaps)	100

The fracture gap size varied from 0 to 25 mm, except one of the early studies by Hulse et al.^35^, where the authors chose a gap size of 60 mm. Although a gap size of 60 mm may be used for construct stability validation as a worst-case scenario, it is not realistic in clinical settings. As shown in Table 2, 10 mm is the most commonly adopted gap size (accounting for 25%) followed by 1 (10%) and 5 mm (10%), while 88% of the gap sizes are within or up to 10 mm. Gap size plays a significant role in the stability of fracture fixation systems: higher gap sizes lead to less stable fixation constructs.^36^ Moreover, the gap size is one of the key influencers on interfragmentary movement (IFM), which is directly linked to callus formation and the bone healing process.^37,38^

**Table 2.** Adopted gap sizes amongst studies on construct testing (*n* = 37).

Standards number	Standards title
ASTM F382-17^39^	Standard specification and test method for metallic bone plates
ASTM F384-17^40^	Standard specification and test methods for metallic angled orthopaedic fracture devices
ASTM F897-19^41^	Standard test method for measuring fretting corrosion of osteosynthesis plates and screws
ASTM F2502-17^42^	Standard specification and test methods for absorbable plates and screws for internal fixation implants
ISO 5836:1988^43^	Implants for osteosynthesis. Bone plates. Specification for holes corresponding to screws with asymmetrical thread and spherical undersurfaces
ISO 9269:1988^44^	Implants for osteosynthesis. Bone plates. Specification for holes and slots for use with screws of 4.5, 4.2, 4.0, 3.9, 3.5 and 2.9 mm nominal sizes
ISO 9585:1990^45^	Implants for osteosynthesis. Bone plates. Method for determination of bending strength and stiffness

Loading regimes include compression,^35–37,46–59^ bending (three or four point)^36,47,52,54,57,59–62^ and torsion testing,^36,48,49,54,60,62^ while most of them are conducted as a combination of compression, bending and torsion to mimic the real load condition of the bones. The end of test criteria are;

(1) Until failure, where the load is applied at a fixed speed of load control or movement control, for example, 26 N/min,^53^ 300 N/s,^55^ 3 mm/min, 5°/min^57^ or cyclic loading conditions.^53^ This is widely adopted as an overall evaluation of the construct, including stress, interfragmentary movement, stiffness, yield load, ultimate load, failure mode and fatigue strength.
(2) Until a fixed load is reached, or for a set number of cycles in the case of cyclic load. This is generally adopted in the finite element analysis approach as boundaries in order to calculate the Von Mises stress distribution. In the cases where it is adopted in laboratory tests, it can assess the construct stiffness as an indicator of construct stability. The maximum cyclic load is dependent upon the anatomic location, and generally varies from 100 to 1500 N. Sod et al.^48^ used an exceptionally high load where they conducted the four-point bending test at a cyclic load between 0 and 7.5 kN because the testing plate is used for equine metacarpal bones.

One more area to take into consideration when setting up the construct test is the plate and screw configuration, such as locking screw or standard screw,^52^ cortical or biocritical screws,^59^ angle of screws,^49,63^ number and space between screws,^37,38,64^ as well as design of the plate fixation construct, such as minimal contact plate,^53^ double plate system,^54^ bridge combination fixation system,^65^ hybrid plate system,^66,67^ helical plate,^68^ screw free plate system,^69^ additional support using bone grafting,^70^ cement,^55^ screw and/or cables.^51,57^

Despite the extensive effort on biomechanical construct evaluation of the fracture fixation plate of different design and materials, unfortunately, these studies do not analyse the debris produced. A search on international testing standards using ISO and ASTM database search engines found there are no standards on testing the plate and screw construct in a way that mimics the application conditions of a fracture fixation system. There are also no standards that describe debris generation tests for plates and screws. This would partly explain the variation in test protocols found in the 37 studies on plate and screw constructs as outlined above. More specifically, for biomechanical testing, there are seven current active international testing standards of fracture fixation plates as shown in Table 3.^39–45^ Of these seven standards listed, six of them focus on dimensional specification, bending and compression testing. There is only one standard on biotribology, which only focusses on fretting corrosion between plate and screws. A solution to this gap is to develop a standard testing protocol for fracture fixation construct testing. With the volume of studies in this field, there needs to be a collaborative effort to bring a standardised method for the industry to use.

**Table 3.** Active international standards for testing of fracture fixation plates, as of May 2020.^39–45^

Standards number	Standards title
ASTM G99-17^71^	Standard test method for wear testing with a pin-on-disc apparatus
ASTM G133-05^72^	Standard test method for linearly reciprocating ball-on-flat sliding wear
ASTM G77-17^73^	Standard test method for ranking resistance of materials to sliding wear using block-on-ring wear test
ASTM G137-97^74^	Standard test method for ranking resistance of plastic materials to sliding wear using a block-on-ring configuration
ASTM G176-03^75^	Standard test method for ranking resistance of plastics to sliding wear using block-on-ring wear test – cumulative wear method
ASTM F732-17^76^	Standard test method for wear testing of polymer materials used in total joint prostheses
ASTM F1714-96^77^	Standard guide for gravimetric wear assessment of prosthetic hip designs in simulator devices
ASTM F2025-06^78^	Standard practice for gravimetric measurement of polymeric components for wear assessment
ASTM F2423-11^79^	Standard guide for functional, kinematic and wear assessment of total disc prostheses
ASTM F2624-12^80^	Standard test method for static, dynamic and wear assessment of extra-discal single level spinal constructs
ASTM F2694-16^81^	Standard practice for functional and wear evaluation on motion preserving lumbar total facet prostheses
ASTM F3047M-15^82^	Standard guide for high demand hip simulator wear testing of hard-on-hard articulations
ISO 14242^83–86^	Implants for surgery. Wear of total hip-joint prostheses
ISO 14243^87–90^	Implants for surgery. Wear of total knee prostheses
ISO 18192^91–93^	Implants for surgery. Wear of total intervertebral spinal disc prostheses
ISO 22622:2019	Implants for surgery. Wear of total ankle-joint protheses
ASTM F1877-16^94^	Standard practice for characterisation of particles
ASTM F561-19^95^	Standard practice for retrieval and analysis of medical devices and associated tissues and fluids
ASTM F2979-14^96^	Standard guide for characterisation of wear from the articulating surfaces in retrieved metal-on-metal and other hard-on-hard hip prostheses
ISO 17853:2011^97^	Wear of implant materials. Polymer and metal wear particles. Isolation and characterisation

## Tribology and debris related testing of fracture fixation plates

Despite the search criteria within focus of tribological testing of fracture fixation plate, it is to note of the 83 papers in this review only three papers within the search criteria are directly linked with tribological issues of fracture fixation plates. This agrees with the biomechanical testing of fracture fixation publications and international testing standards that only limited research have been conducted in this area to date.

**Figure 3.**
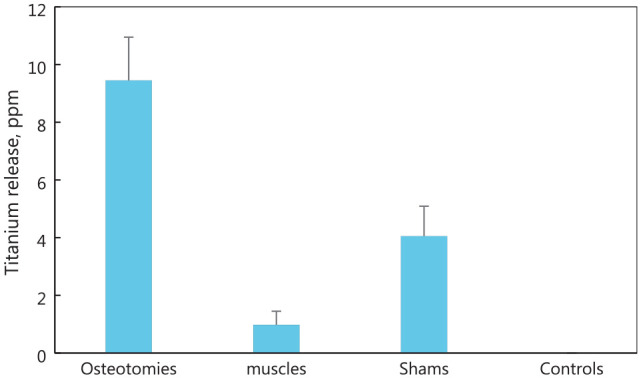
Amount of titanium debris in tissues for each group, reproduced from the study of Mu et al.^99^ Sham refers to a controlled surgery where the plate and screws were extracted immediately after implantation to exclude any surgical procedure caused influences.

**Figure 4.**
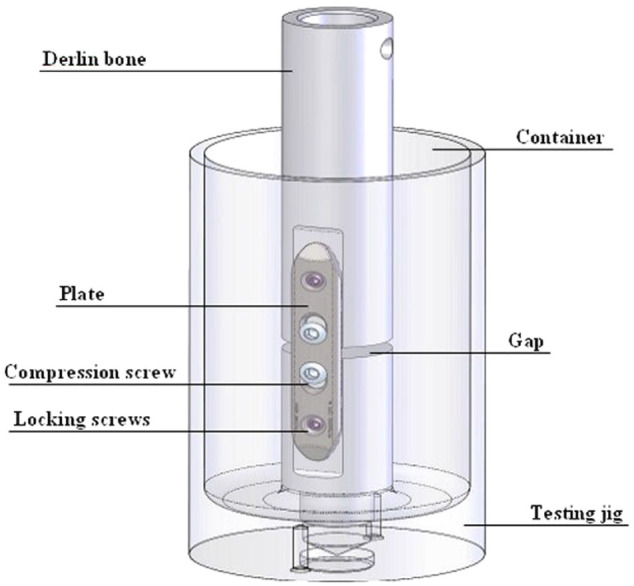
Wear testing jig for fracture fixation by Steinberg et al.,^100^ copyright cleared for reuse.

The first study was in 1978, in which Mutschler et al.^98^ explored the possibility of using lubricants to reduce the friction between the plate and screws when tightening the screws so that the damage of the screw and plate can be minimised during the implantation process. It was found that whilst reducing the friction, the screw force could increase up to 40%.^98^ In addition, the lubricating spray demonstrated no toxicity. However, no further following study was found on using lubricating spray on implants, nor other tribological test following Mutschler et al.’s study. The next study about debris on fracture fixation plates was in 2002 when Mu et al.^99^ investigated the release of titanium debris using a rabbit model. In the study, commercially pure titanium plates and self-tapping screws were implanted into the legs of rabbits in four groups; osteotomy group, where screws and plates was implanted to fix the broken bone and retrieved after 48 weeks; muscles group, where screws and plates were implanted into the muscles without forming a screw and plate construct and retrieved after 48 weeks; sham group, where screws and plate were implanted to fix a broken bone but retrieved immediately after implantation; control group, where no implantation was carried out but tissue was collected for following analysis. Titanium in the tissue were quantitatively studied through atomic absorption spectrophotometer (AES), with the results shown in Figure 3.^99^ Debris was mainly generated from three aspects: during surgical implantation, wear and fretting between the bone, plate and screws when in use and tissue and implant reaction. The percentage of debris generated during these three aspects were approximately 42%, 47.5% and 10.5%, respectively. Nonetheless, during their study, debris generated from implant removal and implant failure was not considered. The most recent and pioneering experimental study on wear of fracture fixation plates was carried out by Steinberg et al.^100^ in 2013, where they quantitatively calculated the amount of debris from the fracture fixation construct in vitro. They designed a testing assembly which contained a fracture fixation construct of the testing plate and screws submerged in buffered saline solution (PBS), shown in Figure 4 ^100^ data-manual-cit = ‘Y’ type = ‘C’>.^100^ The assembly was tested at a load of 300 N for a million cycles. The debris was collected in the container of the assembly and filtered to calculate the amount the debris generated.

Two of the three tribology papers discussed in this review took the collection of debris into consideration. Steinberg et al.^100^ in 2013 calculated the amount of debris using a filtration method. The test was carried out in an enclosed environment, where the debris are collected in the PBS test solution. The authors then filtered the solution through 1 and 0.2 µm filtering paper and measured the weight of the solution before and after each step of filtration. The amount of debris is then worked out through weight deduction. The filtered debris is also sent for observation under optical microscopy to validate the conclusion in terms of which tested plates generated more debris. However, the collected debris from this study could be generated from either the screws, plate, or Delrin rod. The other study was from Mu et al.^99^ where tissue retrieved from the implanted rabbits were placed in a muffle furnace at 600°C for 6 h. The burned ash was then dissolved using concentrated hydrofluoric acid and nitric acid. The debris was then quantitatively analysed using an atomic absorption spectrophotometer. Debris was also histologically studied using optical microscopy and transmission electron microscopy to identify the debris size. However, this quantitative debris analysis method is only applicable to metal plates and screws.

Despite the dearth of debris studies in plates and screws, debris has drawn the attention of surgeons and researchers in the medical field since 1970s. One of the earliest issues that was noticed surrounding debris is implant loosening when Harris et al.^101^ observed extensive localised bone resorption surrounding the total hip replacement in 1976. Since then there has been a high interest, and a series of international testing standards has also been established on orthopaedic implants totalling 18, as shown in Table 4. Those testing standards can be classified into three categories,

- Wear testing methods, which include generic laboratory wear testing methods, for example pin-on-discs, ball-on-flat, block-on-ring on different types of motion such as linear, reciprocating, circular etc. It also includes application specific testing methods such as in spine, hip, knee.
- Characterisation methods. Wear is measured either gravimetrically based on weight changes, or volumetrically based on profilometric assessment of the wear track.
- Debris retrieval and characterisation. This covers particles retrieved from medical devices and its associated tissue and fluid, but also the requirements for particles characterisation. As it defines, a minimum of 100 particles are required to substantially quantify the morphology of debris.

**Table 4.** Active international standards for wear and debris testing on orthopaedics as of May 2020.^71–97^

Authors	Title	Methods to simulate fracture	Type of bones	Considering parameters	Loading mode and test conditions
Hulse et al.^35^	Reduction in plate strain by addition of an intramedullary pin	60 mm gap	Synthetic bones (Polyvinylchloride cylinders)	Two types of fixation: Plate and screw construct; Plate and screw construct with the addition of an IM pin	Axial compression till maximum load of 600 N at a speed of 0.7 cm/s and maintained at 600 N.
					Stress was calculated from strain analysis of the construct.
Abel and Sun^60^	Mechanical evaluation of a new minimum-contact plate for internal fracture fixation	1 mm	Synthetic bones (Sawbones) representing cortical bones with flexural modulus 20.6 GPa; and tensile modulus 27 GPa	Three types of plates: (1) minimal contact plate (MCP), (2) DCP, (3) LC-DCP	Four-point bending carried out on plates according to ISO 9585
					Torsional testing for calculations of torsional stiffness
Borgeaud et al.^46^	Mechanical analysis of the bone to plate interface of the LC-DCP and of the PC-FIX on human femora	0 mm	Human cadaver bone	Two fixation constructs: (1) Internal fixator (PC-Fix); (2) LC-DCP plate	Eccentric load from 0 to 1000 N at a speed of 100 N/s; then unloaded at the same speed.
				Five torque value on screw tightening, 1, 2, 3, 4 and 5 Nm on LC-DCP plate; While a torque of 5 Nm was applied on PC-Fix	Strain at five locations were recorded by strain gauges
Bernarde et al.^47^	An in vitro biomechanical study of bone plate and interlocking nail in a canine diaphyseal femoral fracture model	25 mm gap	Canine femurs	Two fixation constructs: (1) DCP and eight cortical screws, (2) Interlocking nail (IN) with three screws	Five-step testing: (1) continuous eccentric axial compression until maximum 200 N in 60 s, (2) discontinuous eccentric axial compression till maximum 200 N in steps of 40 N at 12 s with a 6 s stabilisation period, (3) continuous bending till maximum load in 80 s, (4) discontinuous bending till maximum 60 N in steps of 6 N at 8 s with 6 s stabilisation period, (5) eccentric axial compression till failure at 5 mm/min for half of each group; OR bending till failure at 5 mm/min
Cheng et al.^102^	Biomechanical evaluation of the modified double-plating fixation for the distal radius fracture	1 mm gap	Simulated bone	Three plating systems: (1) modified double-plating, (2) double plating, (3) single plate	Four sets of axial loads (10, 25, 50 and 100 N), bending (1.0, 1.5, 2.0 and 2.5 Nm) and torsion moments loads (1.0, 1.5, 2.0 and 2.5 Nm)
Benli et al.^103^	Evaluation of bone plate with low-stiffness material in terms of stress distribution	1 mm	simulated bone with Young’s modulus 20 GPa and Poisson ration of 0.3	Three types of plates: (1) stainless steel, (2) Ti plate and (3) materials with low stiffness at various healing stage: (1) 1% healing, (2) 50% healing and (3) 75% healing	Simulated patient weight of 80 kg (equivalent compression pressure of 2.5 MPa) for calculation of stress distribution
Krishna et al.^68^	Analysis of the helical plate for bone fracture fixation	2 mm gap	simulated bones	Three types of plates: (1) straight plate, (2) 90° helical plate, (3) 180° helical plate	Three types of load: (1) compressive load of 150 N, (2) torsional force: 0.05 rad displacement, (3) four point bending loading condition of 0.15 mm displacement transverse direction of the plate and bone construct
Sod et al.^48^	In vitro biomechanical comparison of locking compression plate fixation and limited-contact dynamic compression plate fixation of osteotomized equine third metacarpal bones	0 mm	Equine bone	Two types of plate constructs: (1) 8 hole 4.5 mm LCP, (2) 8 hole 4.5 mm LC-DCP	Four-point bending till failure at a speed of 6 mm/s; Four-point bending cyclic load from 0 to 7.5 kN at 6 Hz till failure; Torsion till failure at a rate of 0.17 rad/s
Windolf et al.^49^	Biomechanical investigation of an alternative concept to angular stable plating using conventional fixation hardware	10 mm gap	Human cadaver bone	Four constructs: Fence elevate LC-DCP, fence non-elevated LC-DCP, LCP, LC-DCP	Sinusoidal axial compression between 100 and 1000 N at 1 Hz for 5000 cycles, till failure; if not continue with torsional sinusoidal loading between +20 and −20 N at 1 Hz for 5000 cycles or till construct failure
Fouad^50^	Effects of the bone-plate material and the presence of a gap between the fractured bone and plate on the predicted stresses at the fractured bone	1 mm gap and 0 mm gap	Simulated bone (finite element analysis) which assumed to be isotropic and uniform with a Young’s modulus of 20 GPa and Poisson’s ratio of 0.3. Callus: Young’s modulus of 0.02 GPa (1% healed, first week), 10 GPa (50% healed, third week), 15 GPa (75% healed, sixth week)	Three types of plating systems: Ti plate, SS plate and new functional graded (FG) plate.	2.5 MPa pressure caused due to body weight (800 N) is used as compressional axial loading. Von Mises compressive stress at the fracture site and bone underneath the plate were calculated and compared at different healing level.
Oh et al.^36^	Effect of fracture gap on stability of compression plate fixation: a finite element study	Different gap sizes: 1 and 4 mm	Synthetic cortical bone cylinder: Young’s modulus of 16.7 GPa with thickness of 2.5 mm and outer diameter of 35 mm	Four experimental testing models depending on the gap size and bone defects: (1) 0 mm, 0%, (2) 1 mm, 100%, (3) 4 mm, 100%, (4) 4 mm, 50%	Four-point bending: at 1 mm/min until construct failure.
				Based on the testing results, FEA was used for further predication of different gap between 0and 4 mm with different bone defects of 0%, 25%, 75% and 100%.	FEA at three load conditions to calculate the peak von mises stresses: (1) Axial compression of 1400 N, (2) torsion with a torque of 5 Nm, (3) four-point bending: 150 Nm bending moment at two ends of the bone-plate construct
Shah et al.^51^	The biomechanics of plate fixation of periprosthetic femoral fractures near the tip of a total hip implant: cables, screws, or both	5 mm gap	Fourth generation synthetic composite bone	Three plating constructs: (1) cables alone, (2) screws alone, (3) cables and screws	Axial compression: preload 50 N, followed by displacement control at 5 mm/min to maximum load of 1000 N. FEA: 1000 N load at the implant femoral ball at simulated femur adduction angle of 15°
Dubov et al.^104^	The biomechanics of plate repair of periprosthetic femur fractures near the tip of a total hip implant: the effect of cable-screw position	5 mm gap	Fourth generation synthetic composite bone	Three plating constructs: (1) cables alone, (2) screws alone, (3) cables and screws	Axial compression: preload 50 N, followed by displacement control at 5 mm/min to maximum load of 1000 N. FEA: 1000 N load at the implant femoral ball at simulated femur adduction angle of 15°
Osterhoff et al.^70^	Medial support by fibula bone graft in angular stable plate fixation of proximal humeral fractures: an in vitro study with synthetic bone	10 mm gap	Synthetic osteoporotic bones	Two fixation constructs: (1) conventional locking plate system, (2) same with (1) but with an additional 6 cm long graft intramedullary inserted	On a shoulder testing device with cyclic force from 50to 125 N; and a device adjusted speed of 300 mm/min for 400 cycles simulating abduction movement from 45° to 60° while lifting simulated arm weight of 3.75 kg at a speed of 5 °/s
Verset et al.^52^	Comparison of the effect of locking vs standard screws on the mechanical properties of bone-plate constructs in a comminuted diaphyseal fracture model	5 mm gap	Ovine tibia bone	Two screws configuration on LCP plate; (1) standard bicortical screws, (2) locking screws	A combination of four-point bending, torsion and axial compression non-destructive test followed by one another.
Avery et al.^61^	A finite element analysis of bone plates available for prophylactic internal fixation of the radial osteocutaneous donor site using the sheep tibia model	4 cm length, 40% circumference with 45° slope end cut	Simulated bone with its density and shape acquisitioned from sheep cadaver tibia	Four different plates: (1) 3.5 mm T-plate of titanium, (2) 2.4 mm T-plate of titanium, (3) 3.5 mm DCP plate of stainless steel, (4) 3.5 mm LCP plate of stainless steel	Four-point bending at 30 Nm (1000 N); Torque at 5 Nm
					Von Mises stresses were calculated. Relatively strengthening effect of bone plates were also calculated by dividing the maximum von Mises stress at the osteotomised control sample by the maximum von Mises stress within the osteotomised region.
Huff et al.^62^	Proximal humeral fracture fixation: a biomechanical comparison of two constructs	10 mm gap at 5 cm distal from humeral head	Synthetic foam/cortical bone for testing and cadaver bone for further validation	Two plating systems: Synthes 3.5 mm proximal humerus LCP and Depuy S3 proximal humerus plate	Bending test: cyclic load to ±5 mm displacement at 1 mm/s for 100 cycles in sagittal plane, followed by same setting in frontal plane, then specimen was tested at 1 mm/s in varus till failure Torsional test: cyclic rotation between 8° of both internal and external submaximal rotation for 100 cycles, followed by 1 °/s externally rotated to failure

Irubetagoyena et al.^53^	Ex vivo cyclic mechanical behaviour of 2.4 mm locking plates compared with 2.4 mm limited contact plates in a cadaveric diaphyseal gap model	20 mm gap	Canine femur	Two types of plates: (1) LCP, (2) LC-DCP	Cyclical compression load: starting with four quasistatic load/unloading cycles between 26 and 260 N; then followed by cyclic loading from 26 to 260 N at 10 Hz for 610,000 cycles. During the cyclic loading, quasistatic loading/unloading were applied at 0 cycles, 10,000 cycles and then every 5000 cycles at a loading rate of 26 N/min.
Chen et al.^54^	Design optimisation and experimental evaluation of dorsal double plating fixation for distal radius fracture	3 mm gap	Simulated bone for FEA model, which is formed with (1) cortical bones of 17 GPa Young’s modules and 0.3 Poisson’s ratio, (2) dense trabecular bone of 1.47 GPa Young’s modulus and 0.3 Poisson’s ratio and (3) low-density trabecular bone of 0.231 GPa Young’s modulus and 0.3 Poisson’s ratio. Synthetic bone for biomechanical testing	L_18_ Taguchi arrays constructed to select the optimal design parameters regarding (1) plate thickness, (2) plate width, (3) screw diameter and (4) number of screws under axial (100 N), bending (1 Nm) and torsion (1 Nm) loads.	Biomechanical testing on selected optimised design at 0°, 30° and 60° angles construct in the following conditions: 10 and 150 N at 5 Hz for 20,000 cycles.
Steinberg et al.^100^	Carbon fibre reinforced PEEK Optima – a composite material biomechanical properties and wear/debris characteristics of CF-PEEK composites for orthopaedic trauma implants	4 mm gap	synthetic bones (delrin rod)	Two types of plates: (1) Titanium plate, (2) CF-PEEK plate	At load of 300 N with *R* = 0.1 for 1 million cycles at 2 Hz
Bagheri et al.^105^	Biomechanical analysis of a new carbon fibre/flax/epoxy bone fracture plate shows less stress shielding compared to a standard clinical metal plate	5 mm gap	synthetic femur bones	Two types of plate: (1) metal plate and (2) carbon/flax/epoxy bone fracture plate	Specimens orientated at 15° of adduction
				Four specimens groups: Stage 1: intact femur alone, Stage 2: intact femur with total hip replacement (THR), Stage 3: 5 mm gap femur with THR and fixation plate, Stage 4: intact femur with THR and fixation plate	Preload at 100 N
					Axial compression tested at average load of 750 N (min 150 N, max 1150 N) at 5 Hz sine waveform
Kainz et al.^55^	Calcium phosphate cement augmentation after volar locking plating of distal radius fracture significantly increases stability	10 mm	Human cadaver radius bone	Two plates: (1) Aptus plate, (2) Synthes plate	Preload at 20 N ant then tested under cyclic compression loading starting from 100 N and increasing 100 N per cycle at a rate of 300 N/s.
				Fracture gap: filled (1) with and (2) without calcium phosphate cement	Samples were tested until failure or when the applied load reaches 1100 N.
Qiao et al.^106^	Bone plate composed of a ternary nano-hydroxyapatite/polyamide 66/glass fibre composite: biomechanical properties and biocompatibility	3 mm gap	Canine femur	Two types of plates both with Ti screws: (1) n-HA/PA66/GF plate, (2) Titanium plate	Four-point bending preload 50 N, at speed of 1 mm/min until construct failure
					Torsion test: at 0.5 °/s until failure
Yavari et al.^67^	Mechanical analysis of a rodent segmental bone defect model: the effects of internal fixation and implant stiffness on load transfer	6 mm	Cadaver rat femurs	PEEK internal fixation plate with porous titanium as bone substitution biomaterials: (1) 120 µm strut diameter, (2) 170 µm strut diameter, (3) 230 µm strut diameter	compression at constant rate of 123 mm/min until the construct failure
Kenzig et al.^107^	A biomechanical comparison of conventional dynamic compression plates and string-of-pearls™ locking plates using cantilever bending in a canine Ilial fracture model	0 mm	Cadaver canine ilial bone	Two constructs: (1) 3.5 mm DCP, (2) 3.5 mm SOP^3^ (string-of-pearls	Preloaded at 5 N, load was then applied at 20 mm/min until failure (acute drop in load)
	No significant biomechanical differences were found between String-of-Pearls™ plate and dynamic compression plate constructs in this simplified cadaveric canine ilial fracture model				
Heyland et al.^64^	Semi-rigid screws provide an auxiliary option to plate working length to control interfragmentary movement in locking plate fixation at the distal femur	10 mm gap and 68 mm above the lateral condyle	Simulated bone that is 80 days postoperative. Axial stiffness 80 N/mm; shear stiffness 40 N/mm	Two types of screws: semi-rigid locking screw (sLS) and rigid locking screws (rLS)	Construct stiffness calculated using predetermined formulae of this model from the author’s previous study.
				Plate working distance: 42, 62, 82 and 102 mm, which corresponds to 1, 2, 3 and 4 empty screws holes; Amount of screws in the configuration	IFM between defined nodes were calculated on applied contact force which corresponds to 45% in the gait cycle of the patient in normal walking from Heller et al.’s^108^ study in 2001.
Kim et al.^56^	Biomechanical study of the fixation plates for opening wedge high tibial osteotomy	10 mm gap	Porcine cadaver tibia	Three plates: (1) Aescular plates, (2) Puddu plates, (3) TomoFix plates	Axial compression under load from 200 to 2000 N, then loaded to failure at speed of 20 mm/min; axial displacement and maximal load at failure were compared; Cyclic load under compression load of 2000 N for 100 cycles, where axial displacements were compared
Batista et al.^57^	Varization open-wedge osteotomy of the distal femur: comparison between locking plate and angle blade plate constructs	15 mm open wedge with/without a 10 mm gap on the medial side	Synthetic bones	Two plates: LCP plate from TomoFix; 95° Angle blade plates from Synthes	Axial compression until maximum load of 1500 N, with a speed of 3 mm/min
				Two fracture gaps: with 10 mm medial gap (FMC); without medial gap (IMC)	Torsion until maximum torque of 7 Nm with a speed of 5 °/min
				Two medical screw configurations: with and without medical cancellous screw in FMC	Comparison on axial and torsional stiffness were made
Samiezadeh et al.^109^	On optimisation of a composite bone plate using the selective stress shielding approach	4 mm gap	Simulated bones	14 different composite bone plate configurations with one metallic plate	Physiological loading, muscle and hip joint reaction forces corresponding to 45% of gait cycle.
Ya-Kui Zhang et al.^58^	Biomechanical effect of the configuration of screw hole style on locking plate fixation in proximal humerus fracture with a simulated gap: A finite element analysis^58^	10 mm gap	Simulated bone; Cortical bone: elastic modulus 12 GPa and Poisson’s ratio 0.3; Cancellous bone: elastic modulus 0.1 GPa and Poisson’s ratio 0.3	Three screw hole configuration at the humerus plate shaft; (1) combi hole, (2) Separate locking and standard hole; (3) locking hole only	Axial loading until 200 N with 50 N increment (four loading steps)
Miramini et al.^37^	The relationship between interfragmentary movement and cell differentiation in early fracture healing under locking plate fixation	1–3 mm gap	Synthetic tibia bones with compressive elastic modulus about 1.5 GPa	Gap size: 1, 3 mm	Compressive load of 100, 150 and 200 N, which represents allowable partial weight bearing after operation.
				Bone plate distance (BPD): 0, 2 and 4 mm	FEA model then established based on the experimental results on IFM obtained from mechanical test to simulate the stem cell differentiation.
				Plate working length (PWL): 30, 65 and 100 mm	
Nourisa and Rouhi^110^	Biomechanical evaluation of intramedullary nail and bone plate for the fixation of distal metaphyseal fractures	3 mm gap	simulated bones	(1) Tibia nail construct and (2) tibia plate construct.	Body weight of 80 kg; Load shared between medial and lateral compartments of tibia plateau by 60% and 40% respectively. Area of load bearing are 468 and 296 mm^2^.
				As well as three types of materials: Ti, SS and Carbon/epoxy	Von Misses stress and axial and shear interfragmental movement is calculated at two load conditions (1) full body weight and (2) 50% body weight
Koh et al.^111^	Multi-objective design optimisation of high tibial osteotomy for improvement of biomechanical effect by using finite element analysis	10 mm wedge	Simulated bone	L27 orthogonal array to study 9 critical to function geometrical dimension of TomoFix tibia plate	Three loading conditions: (1) 150 N with 30° and 90° flection in the FE knee joint on tibia bone, (2) 1150 N axial load, (3) 2500 N compression force corresponding to maximum axial force in gait cycle (for person weight 80 kg)
Tian et al.^112^	An innovative Mg/Ti hybrid fixation system developed for fracture fixation and healing enhancement at load-bearing skeletal site	0 mm; Z-shaped fracture	Rabbit tibia bone	Ti plate with (1) Mg coated screws, (2) Ti screws	Four point bending test on bones recovered after 6 and 12 weeks load applied at 5 mm/min until failure
Sheng et al.^59^	Finite element- and design of experiment-derived optimisation of screw configurations and a locking plate for internal fixation system	3 mm gap	Simulated bone; Cortical bone: elastic modulus 16.8 GPa, Poisson’s ratio 0.3; Cancellous bone: elastic modulus 0.84 GPa, Poisson’s ratio 0.3	Screws configuration of 4 factors and 3 levels being double cortical, single cortical and no screw	A combined loading condition with an axial compression load of 600 N and a torque of 10 Nm at the femur head.
			Artificial sawbones was then used for experimental verification of simulated results	Ten-hole femur diaphyseal plate design parameters; (1) screw hole diameter, (2) screw hole distance. (3) plate width, (4) plate thickness	
Tilton et al.^13^	Biomechanical testing of additive manufactured proximal humerus fracture fixation plates	10 mm gap	low density synthetic bones	Five variants: (1) Conventional proximal humerus LCP, (2) AM reverse engineered anterior to posterior orientation, (3) AM reverse engineered superior to anterior orientation, (4) AM reverse engineered anterior to posterior orientation with solid medial strut, (5) AM reverse engineered anterior to posterior orientation with porous medial struct	Torsional testing at 3.5 Nm under rotational replacement at 0.1 °/s for four cycles followed by axial compression load between 50and 200 N at 0.1 mm/s for four cycles in three configurations: (1) 0°; (2) +20° adduction; (3) −20° adduction followed by cyclic loading at 1 Hz sinusoidal waveform with maximum load increasing 0.25 N/cycles from initial 50 N, until proximal head in contact with superior surface of the shaft. Relative displacement of head shaft and head tuberosity recorded in every 100 cycles.
Baril et al.	Improving greater trochanteric reattachment with a novel cable plate system	13 mm gap	synthetic bones	Two cable plating systems: (1) Zimmer cable ready, (2) novel Y3 Titanium alloy plating system	Customised testing system to simulate two physiological forces on femur implant and greater trochanteric reattachment

**Table 5.** Summary of mechanical laboratory tests (*n* = 37).

Types of bones	Number of studies	Proportion (%)
Synthetic bones	15	38
Simulated bones	12	31
Natural bones	12	31
Total	39 (Two studies have used both synthetic bones and simulated substrate)	100

Only construct testing of the fracture fixation plate is included in this summary.

## Conclusions and future perspectives

Based on the above review, clearly there are emerging designs and materials beginning to be recognised and starting to be used in fracture fixation. However, the current international testing standards are limited on biomechanical evaluation of the plate, for example, bending test. There was an extensive amount of work conducted in the biomechanical evaluation of fracture fixation plates, however, no standard has been established. It is also clear that there are extensive efforts on tribology testing, including debris, in other orthopaedic applications particularly around joint replacement, however, the studies on the tribological characterisation of fracture fixation plates are limited. This highlights both a requirement and an opportunity where these areas of research and testing can be combined to develop a suite of testing methods for fracture fixation devices. The combined testing methods could encompass friction, wear, lubrication and the collection and characterisation of debris, which is becoming increasingly apparent to be important in ensuring the safe development of new materials and design within the field. Emerging needs and requirements for this work to be done that include:

- New biomaterials for fracture fixation plate being developed to improve biomechanical performance. A better understanding is needed of their biotribological behaviour in order to assess the technical readiness level (TRL) of devices made from novel materials.
- Changes in surgical practices in the use of fracture fixation plates has meant that surgeons will often opt for keeping plates in the body rather than remove them once the bone has healed. The long-term effects of keeping these devices in the body may need to be investigated, particularly from the view of debris generation and its effects.
- Medical regulation changes, such as Medical Device Directive (MDD) to Medical Device Regulations (MDR) and the changes to regulatory requirements to demonstrate improved safety and efficacy of new and evolving medical devices.

Future studies could therefore systematically investigate and build an understanding of the biotribology and wear within fracture fixation constructs, as well as the collection and characterisation of the generated debris. Long term, the biological responses of those debris should also be followed. In effort to do so, the authors suggest the development of a suite of tribological testing standards of fracture fixation plates that incorporates;

- Standards on generic pin-on-disc testing (TRL4 and TRL5) as well as other joints can be used to develop standard testing protocols that are fit for purpose for plates and screws.
- A fracture fixation simulator (TRL6) from the current research around construct testing that defines and justifies the testing parameter selection which enables quicker route for product to bedside.
